# Machine-learned analysis of global and glial/opioid intersection–related DNA methylation in patients with persistent pain after breast cancer surgery

**DOI:** 10.1186/s13148-019-0772-4

**Published:** 2019-11-27

**Authors:** Dario Kringel, Mari A. Kaunisto, Eija Kalso, Jörn Lötsch

**Affiliations:** 1 Institute of Clinical Pharmacology, Goethe-University, Theodor-Stern-Kai 7, 60590 Frankfurt am Main, Germany; 2 Division of Pain Medicine, Department of Anesthesiology, Intensive Care and Pain Medicine, University of Helsinki and Helsinki University Hospital, Helsinki, Finland; 3 Fraunhofer Institute of Molecular Biology and Applied Ecology-Project Group Translational Medicine and Pharmacology (IME-TMP), Theodor-Stern-Kai 7, 60590 Frankfurt am Main, Germany; 4 Institute for Molecular Medicine Finland (FIMM), HiLIFE, University of Helsinki, Helsinki, Finland

## Abstract

**Background:**

Glial cells in the central nervous system play a key role in neuroinflammation and subsequent central sensitization to pain. They are therefore involved in the development of persistent pain. One of the main sites of interaction of the immune system with persistent pain has been identified as neuro-immune crosstalk at the glial-opioid interface. The present study examined a potential association between the DNA methylation of two key players of glial/opioid intersection and persistent postoperative pain.

**Methods:**

In a cohort of 140 women who had undergone breast cancer surgery, and were assigned based on a 3-year follow-up to either a persistent or non-persistent pain phenotype, the role of epigenetic regulation of key players in the glial-opioid interface was assessed. The methylation of genes coding for the Toll-like receptor 4 (*TLR4*) as a major mediator of glial contributions to persistent pain or for the μ-opioid receptor (*OPRM1*) was analyzed and its association with the pain phenotype was compared with that conferred by global genome-wide DNA methylation assessed via quantification of the methylation in the retrotransposon *LINE1*.

**Results:**

Training of machine learning algorithms indicated that the global DNA methylation provided a similar diagnostic accuracy for persistent pain as previously established non-genetic predictors. However, the diagnosis can be based on a single DNA based marker. By contrast, the methylation of *TLR4* or *OPRM1* genes could not contribute further to the allocation of the patients to the pain-related phenotype groups.

**Conclusions:**

While clearly supporting a predictive utility of epigenetic testing, the present analysis cannot provide support for specific epigenetic modulation of persistent postoperative pain via methylation of two key genes of the glial-opioid interface.

## Introduction

Persistent pain is a major healthcare problem [1, 2], currently regarded as resulting from neural plasticity including peripheral [3] and central sensitization [4, 5]. The interaction between neurons and glial cells (e.g., microglia and astrocytes) is critical for the initiation and maintenance of persistent pain [6]. Increasing evidence suggests that activation of glial cells contributes to the pathogenesis of persistent pain via neuron-glial interactions [7, 8]. The pro-inflammatory effects of the activation of Toll-like receptors (TLR), positioned at the neuroimmune interface on glia cells, sensory neurons, and other cell types can enhance nociceptive processing leading to exaggerated and unresolved pain [9]. This is mediated in particular by TLR4 that has been shown to induce microglial activation and cytokine production [10]. Moreover, the TLR4 inhibitor (+)-naloxone was able to reverse established neuropathic pain in a nerve injury induced in rats [11].

While (+)-naloxone is inactive at opioid receptors, one of the main sites of interaction of the immune system with persistent pain has been identified as neuro-immune crosstalk at the glial-opioid interface [12, 13]. Indeed, glial cells are involved in opioid actions [14, 15]. For example, the putative toll-like receptor 4 antagonist ibudilast restored morphine-induced antinociception in morphine-tolerant rats [16]. Similarly, minocycline attenuated morphine tolerance in mouse models of neuropathic pain by inhibiting microglial activation [14]. This particular effect on morphine action renders the μ-opioid receptor a key player in glia-opioid crosstalk since the vast majority of current opioid analgesics are mainly μ-opioid receptor agonists.

The involvement of neuroimmune processes in persistent pain is under genetic control [17], suggesting that it may also be under epigenetic control. Indeed, classical epigenetic mechanisms including changes in DNA methylation and histone modifications have been shown to contribute to the development and treatment responsiveness of persistent pain [18]. This was seen at both single gene and global DNA methylation levels. For example, the methylation level of the mu-opioid receptor gene (*OPRM1*) has been associated with acute and chronic postsurgical pain [19]. Similarly, different methylation levels of *LINE1*, which is a retrotransposon of viral provenience spread in approximately half million copies across the human genome [20–22] and therefore used as a marker of global DNA methylation, correlated with different intensity scores of persistent pain [23].

In the present analysis, the methylation status of *TLR4*, *OPRM1*, and *LINE1* was assessed for its association with the persistence of postsurgical pain. DNA samples and pain data were available from a cohort of 1000 women who had undergone breast cancer surgery [24], among whom *n* = 70 patients had developed persistent postsurgery pain, based on ratings acquired up to 36 months after surgery [25]. The previous analysis of the same samples had focused on a role of genetic variants in a selection of pain-relevant genes including the two selected for the present analysis. Indeed, 21 variants in 13 different genes were found to be relevant to the assignment of a patient to either the persistent pain or the non-persistent pain phenotype group [26]. *OPRM1* variants but not *TLR4* variants had been among the relevant genetic markers. Considering that in addition to nucleotide sequence changes, the methylation status can also include the expression of genes, the focus on the glial/opioid intersection in persistent pain was further pursued in the present epigenetic assessments.

## Methods

### Patients and pain phenotype

The study followed the Declaration of Helsinki, and both the Coordinating Ethics Committee (journal number 136/E6/2006) and the Ethics Committee of the Department of Surgery (148/E6/05) of the Hospital District of Helsinki and Uusimaa approved the study protocol. Informed written consent was obtained from each patient. The cohort has been described in detail previously [24, 27]. In brief, 1000 women aged 28–75 years suffering from unilateral non-metastasized breast cancer were enrolled during the preoperative visit. They were treated with breast-conserving surgery or mastectomy, sentinel node biopsy, and/or axillary clearance. Exclusion criteria were neoadjuvant therapy [28] and immediate breast reconstruction surgery. Perioperative analgesia was standardized consisting of preoperative oral acetaminophen, perioperative remifentanil, and postoperative intravenous oxycodone during the first 20 postoperative hours, and ibuprofen or a combination of acetaminophen and codeine during the first postoperative week; no regional anesthesia was used. Adjuvant treatments were given according to international guidelines [27].

As reported previously [25, 29, 30], post-surgical pain intensity was assessed at months 1, 6, 12, 24, and 36 after surgery using numerical rating scale (NRS) ranging from 0 (no pain) to 10 (the most severe pain that can be imagined) [31]. For the diagnosis of persistent pain, NRS data acquired 12–36 months after the surgery were used. As discussed previously [32], due to ongoing adjuvant therapies, this can be considered as more adequately reflecting the clinical setting of breast cancer surgery than the original definition of persistent post-surgical pain, which proposes a cut-off at 2 months [33]. Persistent pain was defined on the basis of NRS ratings as described previously [25], i.e., patients were assigned to the “persistent pain” subgroup if the following conditions applied: NRSmonth36 > 3 and NRSmonth12. month36 > 0 and (NRSmonth36 – NRSmonth24) ≥ 0, whereas patients were assigned to the “non-persistent pain” group if NRSmonth36 ≤ 3 and NRSmonth12. month36 ≤ 3. Applying these criteria to the cohort of 1000 women led to the diagnosis of persistent pain in *n* = 70 patients [25]. For comparison, a similarly sized age and body mass index (BMI)–matched subsample was drawn from the patients who had not developed persistent postsurgical pain.

### Quantification of DNA methylation

DNA methylation levels of CpG sites in *OPRM1*, *TLR4*, and *LINE1* were quantified by means of Pyrosequencing^TM^ assays as used in previous assessments of epigenetic influences on pain [23, 34, 35]. In brief, genomic DNA was extracted from 200 μl of full blood on a BioRobot EZ1 workstation applying the blood and body fluid spin protocol provided in the EZ1 DNA Blood 200 μl Kit (Qiagen, Hilden, Germany) and eluted in a final concentration of 50 ng/μl. Methylation levels were quantified using Pyrosequencing^TM^ assays described elsewhere in full detail [36–39]. The assays were designed to examine the methylation status at (i) four CpG sites in the promoter region of *LINE-1* at bp position − 605, − 593, − 590, and – 583; (ii) six CpG sites in the promoter region of *OPRM1* at bp position − 60, − 50, − 32, − 25, − 18, and – 14; and (iii) four CpG sites in the promoter region of *TLR4* at bp position − 75, − 67, − 58, and − 51, all relative to the start codon. PCR reactions were run on a Mastercycler nexus gradient flexlid device (Eppendorf, Hamburg, Germany) in a 50-μl reaction volume including 5-μl bisulfite-treated DNA, mixed with 0.5 μl MyTaq™ HS DNA polymerase (5 U/μl) (Bioline, Luckenwalde, Germany), 10 μl 5× MyTaq reaction buffer, 0.2 μl of each PCR primer (100 μM), and 34.1 μl HPLC-purified water.

Pyrosquencing™ (Qiagen, Hilden, Germany) took place as described previously [23]. In brief, 50 μl of the PCR templates were processed and purified with the PyroMark Vacuum Prep Worktable (Biotage, Uppsala Sweden) and subsequently annealed to the sequencing primer at 80 °C for 2 min as instructed by the manufacturer. Sequence analysis took place on a PSQ 96 MA System using the PyroMark Gold Q96 Reagents (Qiagen, Hilden, Germany). Pyro Q-CpG methylation software (version 1.0.9) had been used to determine the nucleotide dispensation order. The methylation values represent the mean percentage methylation across all CpG sites, which were measured in duplicate samples within one run. In addition, each sample was measured in two independent runs, which were subsequently averaged. To verify the accuracy of the analysis, each run included control DNA from the EpiTect PCR Control DNA Set (Qiagen, Hilden, Germany) that contained both bisulfite converted 100% methylated, as well as unmethylated, DNA as positive controls and unconverted unmethylated DNA as a negative control.

### Data analysis

Data analysis was performed using the R software package (version 3.4.4 for Linux; http://CRAN.R-project.org/ [40]) on an Intel Core i9® computer running on Ubuntu Linux 18.04.1 64-bit). Epigenetic data comprised methylation status, measured in percentage, of *d* = 6 CpG sites in the *OPRM1* gene, *d* = 4, CpG sites in the *TLR4* gene, and *d* = 4 CpG sites in the *LINE1* retrotransposon, acquired in *n* = 70 patients with persistent pain and *n* = 70 patients assigned to the “non-persistent pain” subgroup. From this 14 × 140-sized data matrix, 15 single values (0.76 %) were missing. Following exploration of the data distribution, which indicated that no transformation was needed, and following a negative test for possible outliers (Grubbs test [41]), gaps in the data space were closed by means of k-nearest neighbors imputation of the missing values. This was done using the R libraries “outliers” (https://cran.r-project.org/package=outliers [42]) and “DMwR” (https://cran.r-project.org/package=DMwR [43]).

The data analysis aimed to identify (i) whether there is an association between the DNA methylation and the persistence of pain, and (ii) which of the assessed genes was implicated in this association. The approach was data-driven and focused on the information about the pain phenotype conferred by the methylation status of the two selected genes or the global methylation status. Specifically, the data analysis followed three main steps. In the first step, basic statistical assessments were performed including assessment of differences in DNA methylation between the two pain phenotype groups, which was done by means of Wilcoxon tests and applying a correction for multiple testing according to Bonferroni [44]. In addition, Spearman correlations [45] were calculated among the components of the matrix of CpG sites and of the original NRS ratings acquired at 1, 6, 12, 24, and 36 months after the surgery. The second step addressed the emergence of structures in the data space of DNA methylation patterns that reflected the known pain phenotype group structure of the patients. The third step of the analysis aimed at associating the methylation status of particular genes with the membership to the pain phenotype groups.

### Methylation pattern analysis using data structure detection

The data space was explored for structures in the DNA methylation patterns that coincided with the known pain phenotype group structure. Unsupervised machine-learning was employed for data structure detection. Specifically, a parameter-free focusing projection method of a polar swarm, *Pswarm*, was used that exploits concepts of self-organization and swarm intelligence. It uses a swarm of intelligent agents called DataBots, which are self-organizing artificial “life forms” that carry vectors of the data. The data space was explored for distance-based structures. Following successful swarm learning on methylation data, rescaled into the range [0,…,100], DataBots carrying items with similar features were placed close to each other in groups on the projection grid. The identification of emergent structures in the learned structure was further enhanced by calculating the distances between data points using the so-called U-matrix [46, 47]. Every value (height) in the U-matrix depicts the average high-dimensional distance of a prototype in relation to all immediate neighboring prototypes regarding grid position. The corresponding visualization technique is a topographical map facilitating the recognition of data structures or clusters. These calculations were performed using the R library “DatabionicSwarm” (https://cran.r-project.org/package=DatabionicSwarm [48]).

For internal validation, data structures were assessed again by means of principal component analysis (PCA) [49]. Specifically, a non-standard implementation of the PCA was used consisting of the “PC-corr” algorithm [50]. By automatically testing various data transformation and analyzing the associated group separations, using quantitative evaluations expressed as *p* value, AUC, and AUPR, assessing any types of normalization and dimension, it permits to find the best results of a PCA. As it calculates various quality measures for every combination of PC, normalization, and centering, it allows the optimal selection of PC for data projection. This analysis was performed using an R script provided with the description of the PC-corr analysis (pccorrv2.R, https://github.com/biomedical-cybernetics/PC-corr_net [50]).

### Association analysis of specific gene methylation sites with pain phenotype group membership

Following the establishment of a data structure supporting a segregation of pain phenotype groups on the basis of the DNA methylation pattern, the contribution of particular genes or CpG sites to the group separation was assessed. Firstly, the results of the PCA performed in the previous analytical step were further explored. This was addressed by calculating the loadings of the CpG sites with the PCA components that explained relevant fractions of the total variance in the data.

Secondly, for internal validation, supervised machine learning methods were applied to narrow the focus on particular CpG sites respectively carrying genes. Specifically, supervised methods were implemented as (i) classification and regression trees [51], (ii) k-nearest neighbors [52], (iii) support vector machines [53], (iv) multinomial regression [54], and (v) naïve Bayesian classifiers [55]. Classification and regression trees use a tree data structure created with conditions on variables (parameters) as vertices and classes (diagnoses) as leaves.

Briefly, tree-structured rule-based classifiers [56] analyze ordered variables *x*_*i*_, such as the present results of methylation analyses [scaled 0 - 100 %], by recursively splitting the data at each node into children nodes, starting at the root node. During learning, the splits are modified such that misclassification is minimized. The Gini impurity was used to find optimal (local) dichotomic decisions as used for the classification and regression tree method (CART) [51]. The calculations were done using the “rpart” function of the similarly named R package (B. Ripley; https://cran.r-project.org/package=rpart). The k-nearest neighbor (kNN) classification [52] provides a non-parametric method that belongs to the most frequently used algorithms in data science although it is one of the basic methods in machine learning. During kNN model building, the entire labeled training dataset is stored while a test case is placed in the feature space in the vicinity of the test cases at the smallest high-dimensional distance. The test case receives the class label according to the majority vote of the class labels of the *k* training cases in its vicinity. The present analyses were performed in *k* = 5 and the Euclidean distance as the default of the R package “KernelKnn” (Mouselimis L, https://cran.r-project.org/package=KernelKnn). Support vector machines are supervised learning methods that classify data mainly based on geometrical and statistical approaches employed for finding an optimum decision surface (hyperplane) that can separate the data points of one class from those belonging to another class in the high-dimensional feature space [53]. Using a kernel function, the hyperplane is frequently selected in a way to obtain a trade-off between minimizing the misclassification rate and maximizing the distance of the plane to the nearest properly classified data point. In the present analysis, a Gaussian kernel with a radial basis was used. The analyses were done using the R library “kernlab” (https://cran.r-project.org/package=kernlab [57]). Multinomial regression provides a method for estimating, from dichotomous or polychotomous data, the probability of occurrence of an event as a function of independent variables [54]. It employs sigmoid data transformation, such as the logit [58], to obtain a linearization, making the data accessible to techniques of multiple regression and its extensions, such as analysis of variance and covariance. The method extends logistic regression to the application on data in which the dependent variable may have a nominal scale with more than two levels, such as in the present three-class problem of three clinical olfactory diagnoses. The present implementation consisted of fitting multinomial log-linear models via neural networks as provided in the R library “nnet” (https://cran.r-project.org/package=nnet [59]). Finally, naïve Bayesian classifiers were used that provide the probability that a data point being assigned to a specific class calculated by application of the Bayes’ theorem [55]. The calculations were done using the R package “klaR” (https://cran.r-project.org/package=klaR [60]).

The analyses were performed in cross-validation runs using 1000 times Monte Carlo [61] to obtain random splits of the original data set into training (2/3 of the data) and test (1/3 of the data) data subsets. This was done using the R library “sampling” (https://cran.r-project.org/package=sampling [62]). For all analyses, the data set was grouped proportionally, with respect to the two pain phenotypes, randomly split into a training data subset (2/3 of the patients) and a test data subset (the remaining 1/3 of the patients). Training of the algorithms with methylation data was performed on the training data subset, and the trained algorithms were then used to identify the group membership of the cases belonging to the test data subset. As main test performance measure, the classification accuracy was used, i.e., the accuracy at which a patient was assigned to her correct pain phenotype group.

To comparatively evaluate the importance of the genes in this task, different combinations of gene-related CpG sites were used, comprising (i) the CpG sites in all three genes, (ii) in each gene separately, and (iii) in combinations of two genes each. Thus, supervised machine learning was used, mainly for knowledge discovery rather than to create a classifier, i.e., a biomarker, for prediction of persistent pain, for which the methylation status in only three genes was judged as unlikely to suffice. The underlying idea was that if an algorithm can be trained with methylation information to identify patients with persistent pain better than by guessing, the information is relevant for the clinical phenotype.

To avoid correct phenotype associations being a result of overfitting rather than based on methylation information, several measures against this weakness of machine learning algorithms were implemented. Firstly, prior to the data analysis, the classification algorithms were tuned with respect to available hyperparameters. For example, the number of *k* in kNN was tested between 3 and 9 and the best performing variant was chosen. Secondly, analyses were performed in cross-validation runs using 1000 times Monte Carlo [61] resampling and data splitting into non-overlapping training and test data subsets. Thirdly, negative control data sets were created by random permutation of the methylation data in the respective training data subsets of each scenario. The expectation was that when trained with random and therefore meaningless data, the algorithm should not perform better than guessing when applied to the association of the pain phenotypes in the test data subsets. Fourthly, five different classifiers were applied to avoid the analysis relying on a single method in which occasional overfitting might have occurred.

## Results

**Fig. 1:**
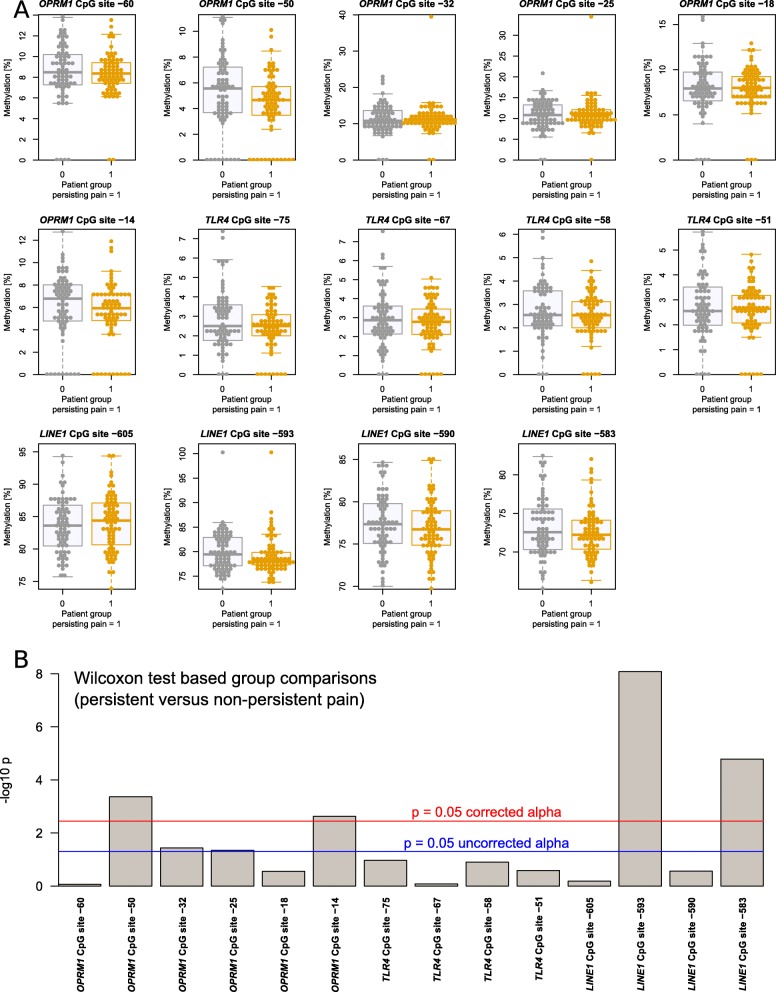
Methylation at *d* = 14 CpG sites located in the *OPRM1* or *TLR4* genes or in the retrotransposon *LINE1* (raw data, for numerical results, see also Table 1). **a** Raw data are shown separately for group membership to the persistent pain or non-persistent pain phenotype groups. The widths of the boxes are proportional to the respective numbers of subjects per group. The quartiles and medians (solid horizontal line within the box) are used to construct a “box and whisker” plot. The whiskers add 1.5 times the interquartile range (IQR) to the 75th percentile or subtract 1.5 times the IQR from the 25th percentile and are expected to include 99.3% of the data if normally distributed. The notches indicate the confidence interval around the median based on median ± 1.57 ∙ IQR/*n*^0.5^. **b** Results of Wilcoxon tests for group differences in the methylation status at each CpG sites. The bars indicate the obtained *p* values, rescaled as –log_10_(*p*). Uncorrected and corrected significance thresholds are shown as horizontal lines. A significant difference is found when the bar exceeds the line. The figure has been created using the R software package (version 3.4.4 for Linux; http://CRAN.R-project.org/ [40])

DNA methylation was quantified from *d* = 6, 4, or 4 CpG sites located in *OPRM1*, *TLR4*, and *LINE1*, respectively, in *n* = 70 patients with persistent pain and in *n* = 70 patients assigned to the “non-persistent pain” phenotype group based on the 3-year follow-up data after breast cancer surgery (Fig. 1a). The demographic data were the following: age 41–73 years (median 58.5 years), BMI 18.4–36.2 kg/m^2^, (median 25.6 kg/m^2^) in the pain group; and age 40–73 years, (median 59 years), BMI 19.9–37.2 kg/m^2^, (median 24.8 kg/m^2^) in the non-pain group. A total of 15 values were missing and were imputed prior to further data analyses. DNA methylation was generally lower in *TLR4* and *OPRM1* than in *LINE1*; specifically, the median methylation across all gene-specific sites and patient subgroups were median [range], *TLR4*: 2.04% [0–7.5%], *OPRM1*: 8.08% [0–39.25%], *LINE1*: 77.78% [65.19–100%].

**Fig. 2:**
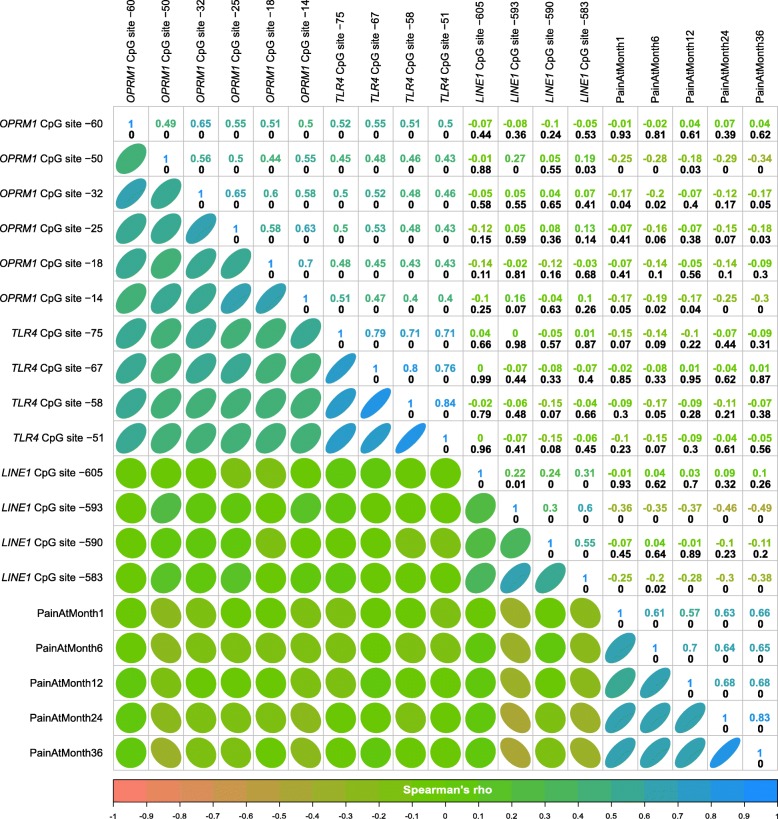
Explorative analysis of the correlations between the methylation status at *d* = 14 CpG sites in *OPRM1*, *TLR4*, or *LINE1* and with the pain ratings acquired between 1 and 36 months after breast cancer surgery. At the lower left part, the correlations are shown as ellipses. The narrower the ellipse is drawn, the higher is the correlation coefficient. Positive correlations are indicated by ellipses directed from the lower left corner to the upper right corner of each cell. Negative correlations are indicated by ellipses drawn in the opposite direction from the upper left to the lower right corner of each cell. Ellipses are colored according to the color code of Spearman’s *ρ* [45] shown at the bottom of the panels. At the upper right parts, the correlations are provided numerically as values of Spearman’s *ρ* (colored). The corresponding *p* values are shown in black numbers below the correlation coefficients; “0” indicates *p* < 1 × 10^−5^. The figure has been created using the R software package (version 3.4.4 for Linux; http://CRAN.R-project.org/ [40]) and the library “corrplot” (https://cran.r-project.org/package=corrplot [63])

Significant differences in DNA methylation between the phenotype groups were observed for CpG sites in *OPRM1* and in *LINE1* (Fig. 1b). DNA methylation tended to be lower in the “persistent pain” phenotype group (Table 1), which agreed with the correlations between *LINE1* methylation and pain ratings which were negative when statistically significant (Fig. 2). In addition, while the correlation structure (Fig. 2) indicated correlations of CpG site methylations within genes, among genes, and between genes and pain ratings, not every variable was correlated with the others. Interestingly, at *OPRM1* CpG sites, the methylation seemed to be significantly correlated with pain ratings only when also correlated with the methylation at *LINE1* CpG sites. By contrast, the methylation at *TLR4* sites displayed the lowest degree of correlation with the methylation at the other genes or with pain ratings.

**Table 1:** DNA methylation observed at CpG sites in the *OPRM1* and *TLR4* genes and in the *LINE1* retrotransposon used to quantify global DNA methylation. Means and standard deviations (SD) are given separately for the “non-persistent pain” and “persistent pain” phenotype groups

CpG site	Non-persistent pain		Persistent pain	
	Mean	SD	Mean	SD
OPRM1 CpG site−60	8.26	2.76	8.3	2.74
OPRM1 CpG site−50	5.5	2.8	3.83	2.39
OPRM1 CpG site−32	11.95	5.21	10.29	3.28
OPRM1 CpG site−25	11.39	4.32	10.12	3.17
OPRM1 CpG site−18	8.1	2.71	7.57	2.99
OPRM1 CpG site−14	6.49	2.7	4.93	3.04
TLR4 CpG site−75	2.77	1.36	2.47	1.39
TLR4 CpG site−67	2.82	1.29	2.82	1.43
TLR4 CpG site−58	2.74	1.08	2.49	1.22
TLR4 CpG site−51	2.8	1.22	2.51	1.21
LINE1 CpG site−605	83.6	4.32	84.07	4.2
LINE1 CpG site−593	81.56	4.75	77.56	1.59
LINE1 CpG site−590	77.47	3.71	76.74	3.07
LINE1 CpG site−583	74.27	4.02	71.66	2.33

### Agreement of DNA methylation patterns with the pain-related phenotype group structure

**Fig. 3:**
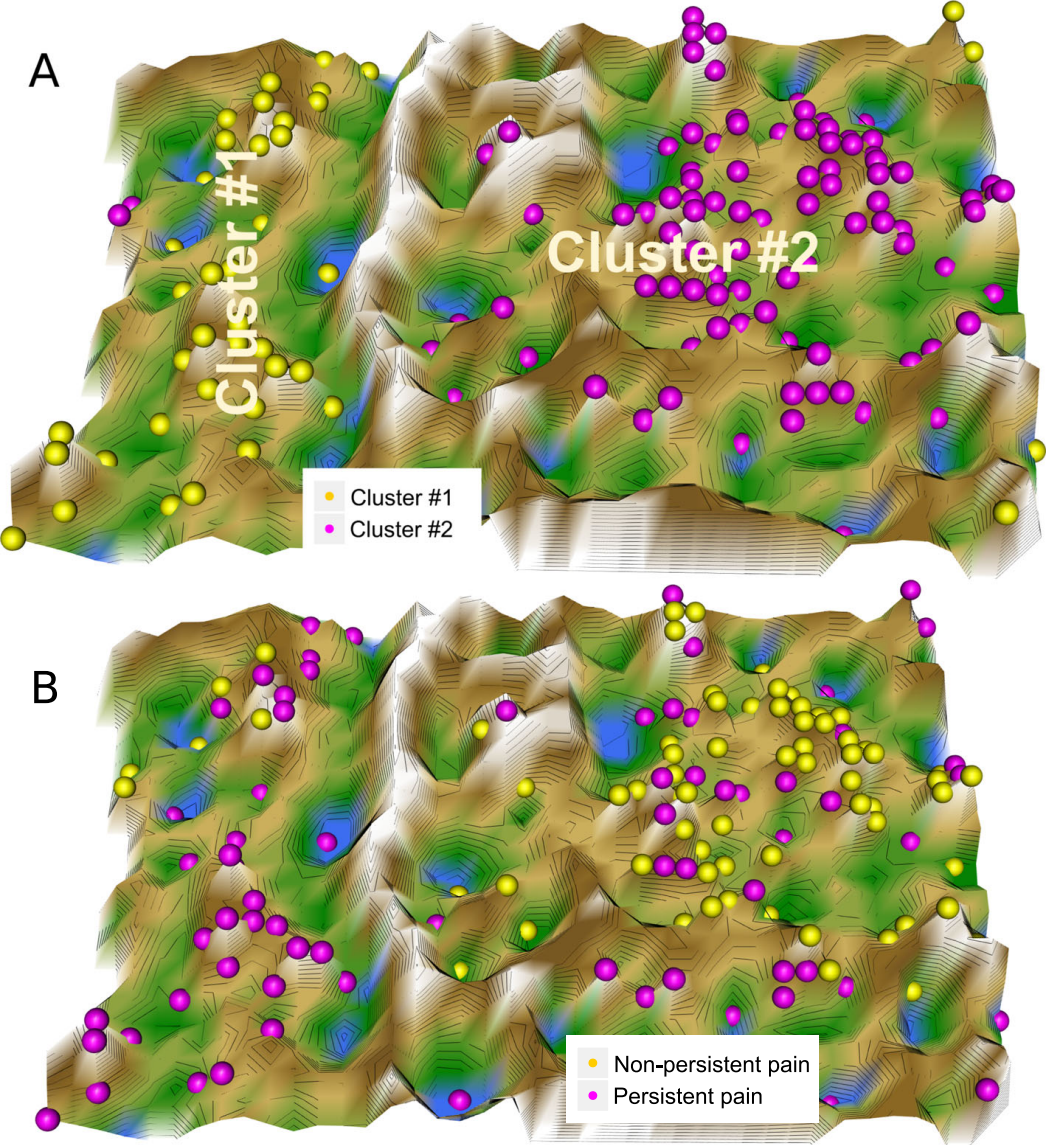
Clustering of subjects based on DNA methylation at CpG sites in *OPRM1*, *TLR4*, and *LINE1*, obtained using unsupervised machine learning. U-matrix visualization of the data structure found via a projection onto a toroid neuronal grid using a parameter-free polar swarm, *Pswarm* consisting of so-called DataBots, which are self-organizing artificial “life forms” that carry vectors of the DNA methylation. **a** The U-matrix visualization was colored as a top view of a topographic map with brown (up to snow-covered) heights and green valleys with blue lakes. Watersheds indicate borderlines between two different clusters. **b** Superimposing the pain phenotype group structure indicated considerable coincidence with the cluster separation, which was supported by a significant *χ*^2^ test of the cross table of clusters versus pain phenotype groups. Please note the different meaning of the coloring of the data points in the two panels, cluster in panel **a** but pain phenotype groups in panel **b**. The figure has been created using the R software package (version 3.4.4 for Linux; http://CRAN.R-project.org/ [40]) and the library “DatabionicSwarm”, https://cran.r-project.org/package=DatabionicSwarm [64])

Swarm intelligence–based data projection followed by U-matrix visualization of the cluster structure supported DNA methylation–derived data structure that reflected the known pain phenotype group structure. Following successful swarm learning, DataBots carrying items with similar features were placed in groups on the projection grid. The distances visualized on the U-matrix indicated a large gap in the data space as a range of large so-called U-heights separating two clusters in which low U-heights indicated that the points are close to each other in the data space, indicating structure in the data set (Fig. 3a). Superimposing onto the cluster structure, the class labeling into patients with persistent or non-persistent pain indicated a separation of the two phenotype groups by the cluster structure (*χ*^2^ = 33.635, df = 1, *p* = 6.649 × 10^−9^).

**Fig. 4:**
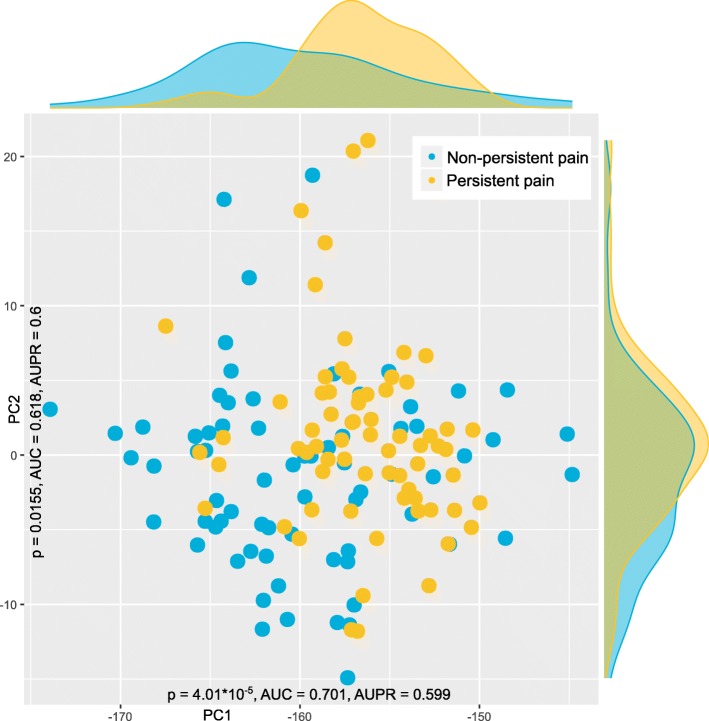
Data structure found in the input space of *d* = 14 CpG methylations acquired from patients with either persistent (*n* = 70) or non-persistent (*n* = 70) pain after breast cancer surgery. The data structure has been obtained by means of data projection principal component analysis on the non-normalized data as suggested by the results of the PC-corr analysis [50]. The PCA plot associated to this analysis shows the sample separation in the first and second component (PC1 versus PC2) yielded the best explained variance for non-normalized, non-centered PCA. The marginal distribution plots show the segregation of the pain phenotype groups along the first principal component. The figure has been created using the R software package (version 3.4.4 for Linux; http://CRAN.R-project.org/ [40]) and the library “ggplot2” (https://cran.r-project.org/package=ggplot2 [65])

Results of the PC-corr analysis (see Additional file 1) supported a non-centered PCA without data transformation as adequate for further group association analyses. Specifically, although the highest amount of variance was explained by the first PC (PC1) when using centering (99.83% versus 99.65% without centering), the non-centered analysis provided higher values of AUC and AUPR indicating slightly better group segregation. Therefore, PCA was done on the non-centered data and further analyses were applied to non-transformed data. Sample segregation along PC1 had *p* value < 0.001, AUC-ROC of 0.7, AUC-PR of 0.6, and explained 99.65% of the variance. Plotting PC1 against PC2, which with non-centered and untransformed data explained the second largest amount of variance indicated a structure in the DNA methylation data that supported the separation of the two pain-related phenotype groups to which the patients had been assigned (Fig. 4).

### Association of specific gene methylation sites with pain phenotype group membership

PCA of the non-centered and non-transformed data provided 11 PCs with eigenvalues exceeding the widely accepted limit of a value of 1 [66]. However, as reported above, the first component explained already > 99% of the total variance (Table 2). It carried loadings mainly from the methylation data of CpG sites located in *LINE1*. The second PC, explaining only 0.15% of the variance, carried mainly loadings related to methylation of CpG sites in *OPRM1*.

**Table 2:** Results of the principal component analysis (PCA) performed at non-normalized and non-centered data, as suggested by the results of the PC-corr analysis [50]. Component loadings of the methylation at CpG sites are shown for PCs with eigenvalues greater than 1. Most of the variance, however, was explained already by the first principal component, PC1

Component	PC1	PC2	PC3	PC4	PC5	PC6	PC7	PC8	PC9	PC10	PC11
Eigenvalue	25066.74	38.56	12.78	8.35	5.67	4.97	4.45	3.5	3.24	2.38	2.13
Explained variance	0.9966	0.00153	0.00051	0.00033	0.00023	0.0002	0.00018	0.00014	0.00013	0.00009	0.00008
Cumulative variance explained	0.9966	0.99808	0.99859	0.99892	0.99914	0.9993	0.99952	0.99966	0.99979	0.99988	0.99997
OPRM1 CpG site−60	− 0.05	− 0.32	0.21	0.1	− 0.43	− 0.05	− 0.11	0.07	− 0.58	− 0.53	0.11
OPRM1 CpG site−50	− 0.03	− 0.3	− 0.03	− 0.2	− 0.36	0.41	0.2	− 0.46	0.36	− 0.18	− 0.41
OPRM1 CpG site−32	− 0.07	− 0.52	0.09	0.22	− 0.25	− 0.58	− 0.06	− 0.16	0.29	0.37	0.15
OPRM1 CpG site−25	− 0.07	− 0.43	− 0.04	0.26	0.19	0.26	0.3	0.64	0.32	− 0.19	0.09
OPRM1 CpG site−18	− 0.05	− 0.36	0.03	− 0.13	0.51	− 0.22	− 0.26	0.03	− 0.19	− 0.05	− 0.65
OPRM1 CpG site−14	− 0.04	− 0.37	− 0.05	− 0.29	0.45	0.21	− 0.13	− 0.38	− 0.05	− 0.09	0.59
TLR4 CpG site−75	− 0.02	− 0.12	0.06	− 0.01	0	0.22	0.05	0.01	− 0.21	0.39	− 0.01
TLR4 CpG site−67	− 0.02	− 0.12	0.09	0.02	− 0.07	0.26	0.01	0.07	− 0.21	0.37	− 0.02
TLR4 CpG site−58	− 0.02	− 0.1	0.08	− 0.01	− 0.08	0.2	0.05	0.09	− 0.22	0.3	− 0.08
TLR4 CpG site−51	− 0.02	− 0.11	0.08	− 0.01	− 0.08	0.22	0.03	0.06	− 0.23	0.34	− 0.01
LINE1 CpG site−605	− 0.53	0.17	0.8	− 0.07	0.12	0.02	0.05	− 0.03	0.16	− 0.04	0.02
LINE1 CpG site−593	− 0.5	− 0.01	− 0.32	− 0.55	− 0.27	− 0.02	− 0.35	0.35	0.12	0.06	0.06
LINE1 CpG site−590	− 0.49	0.08	− 0.28	0.65	0.06	0.25	− 0.37	− 0.22	0	− 0.02	− 0.05
LINE1 CpG site−583	− 0.46	0.01	− 0.31	− 0.04	0.1	− 0.26	0.71	− 0.14	− 0.3	0	− 0.02

**Fig. 5:**
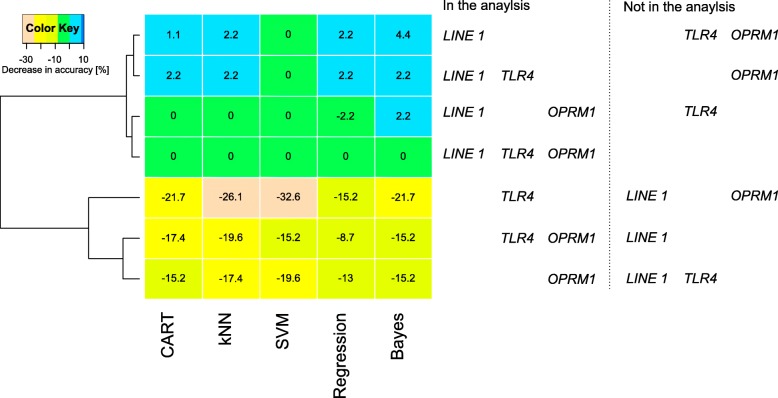
Analysis of the drop in the classification accuracy (Table 3) of five different algorithms (classification and regression trees (CART), *k*-nearest neighbors (kNN), support vector machines (SVM), multinomial regression (“regression”), and naïve Bayes adaptive classification) when the methylation information, originally comprising a total of *d* = 14 CpG sites located in *OPRM1*, *TLR4*, or *LINE1*, was reduced to two or one genes. The numbers indicate the difference in classification accuracy, obtained in several training scenarios of reduced sets of gene-specific CpG islands, to that obtained with the respective algorithm when trained with the full data set. Subsequently, applying hierarchical clustering (Ward [67]) to these differences, a pattern of two groups of the tested scenarios emerged. In the first cluster (top), the accuracy did not change when using a reduced data set for training. By contrast, the accuracy dropped in scenarios included in the second cluster (bottom). The figure has been created using the R software package (version 3.4.4; http://CRAN.R-project.org/ [40]) and the “heatmap.2” function of the R package “gplots” (G.R. Warnes; https://cran.r-project.org/package=gplots)

Supervised machine learning algorisms trained with methylation data succeeded in pain phenotype group association to different degrees depending on the combinations of gene-related CpG sites used for training. When trained with the whole set of gene methylation information, the classification performance ranged between 71% (CART) and 80% (SVM). In all reduced set scenarios where *LINE1* methylation data were included in the training, the classification performance was similar to that obtained when training the algorithms for the complete information about DNA methylation. Moreover, when performing the training only with *LINE1* methylation information, the classification performance was maintained. By contrast, while classification performance was still better than change when including *OPRM1* methylation in the training data subset, it was worse than in the *LINE1* containing scenarios, whereas training with *TLR4* methylation provided a classification not better than chance. Indeed, hierarchical clustering of the decreases in the classification accuracy from the accuracy obtained with the full information identified two clusters for the different scenarios with respect to gene subset inclusion (Fig. 5). One cluster comprised similar classification performance as the full data set and included all scenarios where *LINE1* methylation information was included. By contrast, the second cluster comprised reduced classification performances and included all scenarios without *LINE1*. Importantly, for all scenarios when trained with permuted methylation data, the classification performance of the algorithms was 50%, i.e., like guessing (Table 3).

**Table 3:** Test performance given as accuracy in percentage for the correct assignment to the “persistent pain” patient group (upper part of the table) and as the area under the receiver operator characteristic (AUC ROC, lower part of the table), provided by different types of classifiers obtained using classification and regression trees (CART), *k*-nearest neighbors (kNN), support vector machines (SVM), multinomial regression, and naïve Bayes adaptive classification. The first lines show the obtained accuracy when all *d* = 14 CpG sites were included. Subsequently, several scenarios of reduced sets of gene-specific CpG islands were used for the training of the algorithms, and it was assessed how much leaving out a gene from the training influenced the overall classification accuracy. Furthermore, possible overfitting was accounted for by repeating the training with permuted methylation data created as negative control data sets (AUC ROC omitted). Parameter values were obtained during 1000 runs using Monte Carlo resampling from the original data set. The median of the classification accuracies obtained during the 1000 runs are shown

	CART	kNN	SVM	Regression	Bayes
Classification accuracy					
Training with original data					
All	71.74	73.91	80.43	73.91	73.91
OPRM1	56.52	56.52	60.87	60.87	58.7
TLR4	47.83	47.83	47.83	56.52	50
LINE1	72.83	76.09	80.43	76.09	78.26
OPRM1 and TLR4	54.35	54.35	65.22	65.22	58.7
OPRM1 and LINE1	71.74	73.91	80.43	71.74	76.09
TLR4 and LINE1	73.91	76.09	80.43	76.09	78.26
Training with permuted data					
All	50	50	50	50	50
OPRM1	50	50	50	50	50
TLR4	50	50	50	50	50
LINE1	50	50	50	50	50
OPRM1 and TLR4	50	50	50	50	50
OPRM1 and LINE1	50	50	50	50	50
TLR4 and LINE1	50	50	50	50	50
ROC areas					
Training with original data					
All	79.35	73.91	82.61	80.72	80.15
OPRM1	59.78	53.31	61.81	69.19	63.94
TLR4	53.97	54.06	54.49	61.34	55.10
LINE1	79.02	76.09	84.31	82.23	79.96
OPRM1 and TLR4	58.60	53.07	67.86	72.40	61.81
OPRM1 and LINE1	79.49	73.91	81.47	80.15	81.66
TLR4 and LINE1	79.11	78.26	85.07	82.61	80.72

## Discussion

Supporting the hypothesis of an epigenetically modulated component of persistent pain after breast cancer surgery, the degree of global methylation quantified at four CpG sites in the retrotransposon *LINE1* was correlated with the pain ratings acquired from 6 to 36 months after breast cancer surgery. The agreement of the data structure emerging in the degrees of gene methylation, detected by applying unsupervised data analysis methods including machine learning with the a priori classification of the patient cohort into a “persistent” and a “non-persistent pain” phenotypic subgroup, selected as representing the extreme pain-related phenotypes from a cohort of 1000 women treated surgically for breast cancer, provided further support to the hypothesis of an epigenetically modulated component of persistent pain after breast cancer surgery. Additional support for this hypothesis was provided by the ability of five different supervised algorithms, including machine learning, to assign a patient to the correct pain-phenotype subgroup based on the training with the information about the DNA methylation. This succeeded with an even higher accuracy than that obtained with a recently proposed rule-based classifier, created in the same cohort from demographic, psychological, and pain-related, but not genetic or epigenetic parameters, which provided a sensitivity and specificity of 82.4 and 55.6%, respectively, and an accuracy of 69% [25] to identify patients with persistent pain. Please note that the then used definition of persistent pain reports NRS ≥ 4 as a criterion, which is the same as the presently used NRS > 3 criterion, considering that the NRS is integer-scaled. For example, the class assignment based on the epigenotypes, achieved using support vector machines (Table 3), provided a sensitivity and specificity of 69.6 and 91.3%, respectively, and an accuracy of 80.4%. By contrast, when training the artificial intelligence with permuted epigenetic information, its classification performance dropped to the level of guessing, supporting that the classification success was not due to overfitting. This is promising with respect to using epigenetic information as a biomarker for pain persistence. Moreover, a combination of several kinds of information, genetic, epigenetic, clinical, demographic, i.e., a combination of the previously and presently reported positive results seems a promising approach to a better biomarker, which, however, would greatly exceed the present report.

An association of the global DNA methylation status with persistent pain has previously been suggested by preclinical and clinical research. For example, in rats with post nerve injury pain, the genome-wide DNA methylation in the prefrontal cortex and in T cells was found to differ among pain phenotype groups, based on analysis of methylated DNA immunoprecipitation followed by hybridization to microarrays [68]. Among the all measured probes, methylation was decreased in 14,298 probes and increased in 9088 in animals with a spared nerve injury. Also in rats, nerve injury was shown to cause DNA methylation changes at 8% of the CpG sites across the whole genome, with prevailing hypomethylation outside of CpG islands, based on digital restriction enzyme analysis of methylation in dorsal root ganglion tissue [69]. Nerve injury caused DNA methylation changes at 8% of CpG sites with prevailing hypomethylation outside of CpG islands, in introns, intergenic regions, and repetitive sequences. However, it caused more gains of methylation in the spinal cord and prefrontal cortex. In humans, global DNA methylation differed significantly between patients with low back pain and controls, based on enzyme-linked immunosorbent assays of white blood cells [70]. Furthermore, in outpatients treated in a pain unit of tertiary care, significant positive correlation between the methylation of CpG sites located in LINE1 and pain ratings have been reported [23]. Taken together, the association of higher or lower pain with higher or lower global DNA methylation has been inconsistently reported. While in the present samples, higher methylation of *LINE1* was correlated with lower pain ratings, in a previously analyzed mixed cohort of outpatients of a tertiary care pain treatment unit, the correlation had been positive [23]. In that study, a methylating effect of opioid treatment had been proposed based on higher methylation levels in opioid treated than in non-opioid treated pain patients. This effect has been reproduced independently [71]. Nevertheless, the global methylation levels of in median 80% in that study [23] agreed with the presently observed levels. In the present study, the patients had received a standardized perioperative treatment with opioids and none of the patients had been taking opioid medication either before or after surgery apart from codeine during the first postoperative week, a possible DNA methylation effect of opioids would not have been restricted to one of the subgroups and is therefore also unlikely to have caused the observed subgroup differences; neither can it explain the different direction of the correlation with pain ratings. In the present assessments, lower *TLR4* methylation would have provided a plausible association with higher pain intensity; however, *TLR4* methylation was not correlated with global or *ORRM1* DNA methylation.

While the methylation of *OPRM1* sites, as far as significantly associated with higher pain ratings, apparently provided support for epigenetic control of persistent pain after breast cancer surgery via neuro-immune crosstalk at the glial-opioid interface, supervised machine-learned analyses clearly contradicted this interpretation about the role of *OPRM1* methylation versus global DNA methylation. The methylation at single genes was not needed to assign a patient to the correct pain-phenotype subgroup, hence, neither *OPRM1* nor *TLR4* methylation provided relevant information to train artificial intelligences to perform this phenotype group assignment. For example, the classification accuracy of support vector machines remained completely unaffected at 80.43 when omitting *OPRM1* methylation, *TLR4* methylation, or both, from the training, whereas it dropped by 15% or more when omitting *LINE1* methylation (Table 3). Thus, the partly correlated methylations in *OPRM1* probably just followed the global DNA methylation status reflected in *LINE1*, without indication that they represented a gene-specific mechanism of the regulation of postoperative persistent pain.

The interpretation that the observed epigenetic associations with the development of persistent pain after breast cancer surgery have to be attributed to the global methylation not reflecting a specific regulation in *OPRM1* is unlikely to change if more than six CpG sites in *OPRM1* were analyzed. Reanalyzing previously published data [23] of the methylation at 22 CpG sites in *OPRM1* indicated that DNA methylation was highly positively correlated among all 22 sites, with a median value of Spearman’s *ρ* of 0.552 (range *ρ* = 0.256–0.747) and a median significance level of *p* = 6.11 × 10^−15^ (range *p* = 7.34 × 10^−47^–0.00077). Hence, it seems unlikely that the analysis of more sites within *OPRM1* would have changed the present results. However, an epigenetic control via *OPRM1* or *TLR4* remains possible via histone modulation, which was not assessed in the present study. It is known to play a role in pain-related human phenotypes such as the bladder pain syndrome [72] or as one of the mechanism via which valproate is effective in the management of diabetic neuropathy [73]. Furthermore, since DNA methylation is tissue specific [74], a negative result obtained in DNA extracted from blood cells does not exclude an epigenetic modulation via *TLR4* or *OPRM1* in the central nervous system. Finally, the hypothesized epigenetic control of neuroimmune crosstalk in persistent pain remains a possibility via further genes involved in the glial-opioid interface [17].

Using a case-control approach and drawing from the patients belonging to the non-persistent pain group, a similarly sized sample as the subgroup of patients with persistent pain introduced some limitations in the analysis via oversampling of cases versus controls. Specifically, the present sample was taken to compare the epigenotypes of the subjects with the extreme pain phenotypes of interest, while intermediate phenotypes were omitted. This is a standard design which has several variants such as using matched pairs. Therefore, the numerical values of the pain phenotype group association accuracy may require revision when applied to a non-selected cohort. Hence, the reported classification performances of the algorithms are not presented as a proposal of a diagnostic tool but have been used in a knowledge-discovery manner, aimed at identifying DNA locations within preselected genes where the degree of methylation is distinctive between extreme pain phenotypes after breast cancer surgery. In addition, in a larger sample, more cases available for algorithm training may lead to improved classification performance. However, while machine-learning often unveils its power in so-called big data, its definition is purely methodological as it is referred to as a set of methods that can automatically detect patterns in data and then use the uncovered patterns to predict or classify future data, to observe structures such as subgroups in the data or to extract information from the data suitable to derive new knowledge [75–77]. This meets exactly the present application of machine learning.

The limited, hypothesis-driven selection of two groups of genes, rather than performing a more comprehensive quantification of genome-wide DNA methylation in many other genes relevant to pain and its persistence, further emphasizes the knowledge-discovery focus of the present analysis. The present analysis was performed in a collaborative EU project about persistent pain and it explicitly set the “focus on glial-opioid receptor interface” (project “D” in Table 1 in [78]). The two gene families were exclusively named in the project reported here. More comprehensive assessments of the role of DNA methylation in persistent pain may use other candidate gene approaches in the future. At least 540 genes have been so far demonstrated to be relevant to pain [79–82]. Those approaches may also address the DNA methylation across the whole genome without a restriction to prior knowledge about pain-relevant sites.

## Conclusions

Using information on the methylation of CpG sites, machine-learned analysis indicated that the epigenotypes provide useful information for the allocation of the patients to either a “persistent pain” or “non-persistent pain” phenotype group in a 3-year follow-up after breast cancer surgery. The global DNA methylation, quantified at CpG sites located in the retrotransposon *LINE1*, provided a similar diagnostic accuracy for persistent pain as the previously established non-genetic predictors, based on a single DNA sample. By contrast, the methylation of *TLR4* or *OPRM1* genes could not contribute further to the allocation of the patients to the pain-related phenotype groups. Therefore, the present analysis cannot provide support for specific epigenetic modulation of persistent postoperative pain via methylation of two key genes of the glial-opioid interface. Finally, although the findings regarding the focused hypothesis of a regulation of persistent pain via methylation of *OPRM1* and *TLR4* genes were negative, an accuracy of group assignment approaching 80% by using global epigenetic information encourages further exploration of DNA methylation as a possibly important component of a future biomarker for risk of persistent pain. The analysis emphasizes the need to include a marker for global DNA methylation in epigenetic analyses to prevent that an effect, such as a group difference, being wrongly attributed to the methylation of a specific gene.

## Supplementary information

**Additional file 1.** Supplemental information includes a table of results of a PC-corr analysis of the data in Microsoft Excel format (PCcorr_results.xlsx).

## Data Availability

The patients’ consent does not include public availability of source data or materials.

## Abbreviations

DNA: Deoxyribonucleic acid
LINE1: LINE1 retrotransposable element 1
NRS: Numeric rating scale
OPRM1: Opioid receptor mu 1
PCA: Principal component analysis
TLR4: Toll-like receptor 4
